# Supplementary material for: Visualization of liquid-liquid phase transitions using a tiny G-quadruplex binding protein
Source: Nat Commun. 2025 Sep 29;16:8578. doi: 10.1038/s41467-025-63597-7 (PMC12480911; doi:10.1038/s41467-025-63597-7)
Supplement: Supplementary file 3 — Description of Additional Supplementary Files [file 41467_2025_63597_MOESM3_ESM.pdf]

**Description of Additional Supplementary Files**

File Name: Supplementary  Movie 1
Description: The video shows the formation of a large condensedlike SERF2-TERRA10 rG4 complex structure and a few small oligomeric structures during a 0.5-microsecond all-atom MD simulation. The cartoon representation of SERF2 and RNA molecules is shown in orange and blue, respectively, in the VMD program. The black spheres represent the center of mass of each SERF2 molecule in the system. Water, ions, and crowding molecules are removed from the movie generation in VMD.
