# Supplementary material for: Visualization of liquid-liquid phase transitions using a tiny G-quadruplex binding protein
Source: Nat Commun. 2025 Sep 29;16:8578. doi: 10.1038/s41467-025-63597-7 (PMC12480911; doi:10.1038/s41467-025-63597-7)
Supplement: Supplementary file 5 — Reporting Summary [file 41467_2025_63597_MOESM5_ESM.pdf]

Corresponding author(s): *Double-anonymous peer review submissions: write DAPR and your manuscript number here instead of author names.*

Last updated by author(s): Aug 12, 2025

# Reporting Summary

Nature Portfolio wishes to improve the reproducibility of the work that we publish. This form provides structure for consistency and transparency in reporting. For further information on Nature Portfolio policies, see our Editorial Policies and the Editorial Policy Checklist.

## Statistics

For all statistical analyses, confirm that the following items are present in the figure legend, table legend, main text, or Methods section.

| n/a | Confirmed | |
|---|---|---|
| [ ] | [x] | The exact sample size (*n*) for each experimental group/condition, given as a discrete number and unit of measurement |
| [ ] | [x] | A statement on whether measurements were taken from distinct samples or whether the same sample was measured repeatedly |
| [ ] | [x] | The statistical test(s) used AND whether they are one- or two-sided<br>*Only common tests should be described solely by name; describe more complex techniques in the Methods section.* |
| [x] | [ ] | A description of all covariates tested |
| [ ] | [x] | A description of any assumptions or corrections, such as tests of normality and adjustment for multiple comparisons |
| [ ] | [x] | A full description of the statistical parameters including central tendency (e.g. means) or other basic estimates (e.g. regression coefficient) AND variation (e.g. standard deviation) or associated estimates of uncertainty (e.g. confidence intervals) |
| [ ] | [x] | For null hypothesis testing, the test statistic (e.g. *F*, *t*, *r*) with confidence intervals, effect sizes, degrees of freedom and *P* value noted<br>*Give P values as exact values whenever suitable.* |
| [x] | [ ] | For Bayesian analysis, information on the choice of priors and Markov chain Monte Carlo settings |
| [x] | [ ] | For hierarchical and complex designs, identification of the appropriate level for tests and full reporting of outcomes |
| [x] | [ ] | Estimates of effect sizes (e.g. Cohen's *d*, Pearson's *r*), indicating how they were calculated |

*Our web collection on statistics for biologists contains articles on many of the points above.*

## Software and code

Policy information about availability of computer code

| | |
|---|---|
| Data collection | NMR data were acquired using Bruker 600MHz, 800MHz equipped with TopSpin 4.1.4; Analytical ultra centrifugation data recorded using Beckman Optima Xl-I provided in-built program (program: ProteomeLab XL-I); Protein and oligonucleotide concentration measurement was done using UV-1900 (program: UV Probe 2.70), Microscope images are analyzed using FIJI ImageJ 1.53t; Fluorescence polarization data are collected using a Tecan INFINITE M1000 Pro plate reader (program: i-Control ); Circular dichroism data acquired using HORIBA JASCO (program: SpectraManager), Static fluorescence, absorbance and anisotropy data are acquired by TECAN infinite M1000 Pro plate reader (program: i-control), HORIBA Fluoromax (program: FluorEssence v3.9) and Cary Eclipse using instruments in-built programs, Size-exclusion data were collected using GE AKTA Pure (UNICORN 7.0), Liquid droplet imaging was done using Nikon Ti2-E motorized inverted microscope controlled by NIS Elements software; immunofluorescence imaging was done a Leica TCS SP8 STED 3X confocal microscope with bulit-in LAS X program or Thunder imaging systems. MD simulation data were collected on High-Performance Research Computing facility at National Brain Research Centre (NBRC), India. |
| Data analysis | NMR data analysis by Bruker TopSpin 4.1.1, NMRFAM-Sparky 1.470; Molecular visualization was done using ChimeraX 1.3, PyMOL (academic license) and Discovery Studio Visualizer; Statistical analysis was done using GraphPad Prism 10.1.2 and OriginPro 2020; Analytical ultra centrifugation analysis was done using Ultrascan III, CD absorbance using JASCO program, UV absorbance and fluorescence data fitting were done using OriginPro or GraphPad Prism. MD data were analyzed using gromacs and MDanalysis programs. |

For manuscripts utilizing custom algorithms or software that are central to the research but not yet described in published literature, software must be made available to editors and reviewers. We strongly encourage code deposition in a community repository (e.g. GitHub). See the Nature Portfolio guidelines for submitting code & software for further information.

## Data

Policy information about availability of data

All manuscripts must include a data availability statement. This statement should provide the following information, where applicable:

- Accession codes, unique identifiers, or web links for publicly available datasets
- A description of any restrictions on data availability
- For clinical datasets or third party data, please ensure that the statement adheres to our policy

NMR resolved solution structure of human SERF2 is deposited to Protein Data Bank under accession code 9DT0. The PDB entry is deposited on 2024-09-30, and currently "on hold for release". The NMR assignments are available under the Biological Magnetic Resonance Data Bank or BMRB entry code 52141.

## Research involving human participants, their data, or biological material

Policy information about studies with human participants or human data. See also policy information about sex, gender (identity/presentation), and sexual orientation and race, ethnicity and racism.

| | |
|---|---|
| Reporting on sex and gender | n/a |
| Reporting on race, ethnicity, or other socially relevant groupings | n/a |
| Population characteristics | n/a |
| Recruitment | n/a |
| Ethics oversight | n/a |

Note that full information on the approval of the study protocol must also be provided in the manuscript.

# Field-specific reporting

Please select the one below that is the best fit for your research. If you are not sure, read the appropriate sections before making your selection.

- [x] Life sciences
- [ ] Behavioural & social sciences
- [ ] Ecological, evolutionary & environmental sciences

For a reference copy of the document with all sections, see nature.com/documents/nr-reporting-summary-flat.pdf

# Life sciences study design

All studies must disclose on these points even when the disclosure is negative.

| | |
|---|---|
| Sample size | Sample sizes for cellular, biophysical and biochemical experiments were based on accepted standards for the field. |
| Data exclusions | No data were excluded from this study |
| Replication | Independent replicates were obtained for all biochemical experiments included in the study and are typically presented as mean values or comparisons of means. Measurements were repeated at least two times and all were reproducible. |
| Randomization | Allocation was not randomized, reported values are measured using instruments/techniques that were not thought to be subject to interpreter bias. |
| Blinding | No blinding was used, the instruments/techniques that were used to gather data were not thought to be subject to interpreter bias |

# Reporting for specific materials, systems and methods

We require information from authors about some types of materials, experimental systems and methods used in many studies. Here, indicate whether each material, system or method listed is relevant to your study. If you are not sure if a list item applies to your research, read the appropriate section before selecting a response.

## Materials & experimental systems

| n/a | Involved in the study |
| --- | --- |
| ☐ | ☒ Antibodies |
| ☐ | ☒ Eukaryotic cell lines |
| ☒ | ☐ Palaeontology and archaeology |
| ☒ | ☐ Animals and other organisms |
| ☒ | ☐ Clinical data |
| ☒ | ☐ Dual use research of concern |
| ☒ | ☐ Plants |

## Methods

| n/a | Involved in the study |
| --- | --- |
| ☒ | ☐ ChIP-seq |
| ☒ | ☐ Flow cytometry |
| ☒ | ☐ MRI-based neuroimaging |

# Antibodies

| | |
| --- | --- |
| Antibodies used | anti-SERF2 (Proteintech, 11691-1-AP), mouse anti-G3BP1 (BD Biosciences, 611127), mouse anti-Fibrillarin (Boster Bio, M03178-3), mouse anti-Nopp140 (Santa Cruz, sc-374033), mouse anti-USP10 (Santa Cruz, sc-365828), mouse anti-FUS (Thermo Fisher, 50-554-337), mouse anti-BG4 (Absolute antibody, Ab00174-1.1), mouse anti-TIA1 (Santa Cruz, sc-398372), and mouse anti-eIF2α (Santa Cruz, sc-133132), goat-anti-rabbit secondary Alexa Fluor Plus 488 (Thermo Scientific, A32731), and goat-anti-mouse secondary Alexa Fluor 647 (Thermo Scientific, A21235), goat-anti-mouse secondary Alexa Fluor 488 (Invitrogen, A11001) |
| Validation | All antibodies are validated suitable by the manufacturer for immunohistochemistry and western blot |

# Eukaryotic cell lines

Policy information about cell lines and Sex and Gender in Research

| | |
| --- | --- |
| Cell line source(s) | HEK 293T/17 cells (ATCC® CRL-11268™) used in this study are derived from human embryonic kidney tissue, HeLa Kyoto cells (CVCL_1922) derived from human papillomavirus-related cervical adenocarcinoma, BJ fibroblast cells established from skin taken from normal foreskin from a neonatal male, and U2OS cells derived from a sarcoma of the tibia of a 15-year-old female osteosarcoma patient and was purchased from Sigma-Aldrich (Cat. no. 92022711-1VL). |
| Authentication | Cell authentication was done by testing sterility and mycoplsma contamination. |
| Mycoplasma contamination | We confirm that all cell lines tested negative for mycoplasma contamination. |
| Commonly misidentified lines (See ICLAC register) | n/a |

# Plants

| | |
| --- | --- |
| Seed stocks | n/a |
| Novel plant genotypes | n/a |
| Authentication | n/a |
